# Protein Lactylation Modification and Proteomics Features in Cirrhosis Patients after UC-MSC Treatment

**DOI:** 10.3390/cimb45100532

**Published:** 2023-10-18

**Authors:** Ye Xie, Ying Li, Jia Yao, Xiaojing Song, Haiping Wang, Jianjun Zhang, Xun Li

**Affiliations:** 1 The First Clinical Medical College, Lanzhou University, Lanzhou 730000, China; 2 General Surgery Department, The First Hospital of Lanzhou University, Lanzhou 730000, China; 3 Key Laboratory of Biotherapy and Regenerative Medicine of Gansu Province, Lanzhou 730000, China; 4 Hepatopancreatobiliary Surgery Institute of Gansu Province, Lanzhou 730000, China

**Keywords:** liver cirrhosis, mesenchymal stem cell, cell therapy, lactylation modification, proteomics analysis

## Abstract

Umbilical cord mesenchymal stem cell (UC-MSC) therapy improves liver function in liver cirrhosis patients. This study aimed to elucidate the therapeutic mechanism underlying cell therapy by analyzing changes in the modification and expression of proteins 1 month post-treatment with UC-MSCs. This prospective study included 11 cirrhosis patients who received MSC injection. The laboratory indexes before and after treatment were collected to evaluate the clinical treatment effect of UC-MSCs, and the protein expression and lactylation modification in the liver were comprehensively revealed. Meanwhile, weighted gene co-expression network analysis was used to analyze the co-expression protein modules and their relationship with clinical features. The patients with liver cirrhosis showed an improvement trend after receiving UC-MSC treatment; specifically, the liver protein synthesis function was significantly improved and the coagulation function was also significantly improved. Proteomics combined with lactic acid proteomics revealed 160 lysine lactylation (Kla) sites of 119 proteins. Functional analysis showed that the lactylation-modified proteins were enriched in the pathway of glucose and other substances’ metabolism, and many key enzymes of glycolysis and gluconeogenesis were lactated. UC-MSC therapy has a certain clinical effect in the treatment of liver cirrhosis and may act by regulating material metabolism, because the lactylation protein points to energy metabolism.

## 1. Introduction

Lactylation modification is a new post-translational modification protein. In 2019, the team of Zhang et al. first discovered a post-translational modification method with gene transcription regulation function in histones [1]. At the same time, Zhang et al. proved that histone lactylation is the product of glycolysis, which is regulated by the level of intracellular lactate in a dose-dependent manner, establishing an inseparable relationship between the level of lactate and histone lactylation [1]. The regulation of histone lactate on gene expression was verified in macrophages, which can affect the mutual transformation of macrophage pro-inflammatory or anti-inflammatory phenotypes [1,2]. In physiological conditions, researchers also found that histone lactylation is ubiquitous and plays an important role in epigenetic regulation [3]. In recent years, with the development of high-sensitivity mass spectrometry technology, it has recorded the function of histone and non-histone lactylation on gene transcription regulation, revealing its important role in tumor, inflammation and regeneration [4,5]. In addition, high-throughput protein analysis based on mass spectrometry can serve as a supplement to protein modification omics, analyzing the composition, structure, expression, modification status, and interactions and connections between proteins as a whole [6]. Multi-proteomic analysis combining proteins and modifications is increasingly being applied in revealing the mechanisms of disease occurrence and development [7,8,9].

The liver is the center of human glucose metabolism. It is an important biological process to regulate human blood sugar by transforming monosaccharides into liver glycogen reserves in the liver. When the liver glycogen reserve decreases, the liver can convert some fat, protein, lactic acid, etc. into glycogen through gluconeogenesis. When liver fibrosis occurs and liver cells are damaged, the balance of glucose metabolism is disturbed. First, static liver stellate cells are activated into muscle fibroblasts. During this process, aerobic glycolysis is enhanced, leading to increased lactic acid [10,11]. Then, the gluconeogenesis function of liver cells is abnormal, and the conversion rate from lactate to glycogen is slow [12,13]. Finally, after the formation of liver cirrhosis, the abnormal hepatic lobules and tissues in the liver are hypoperfused to form an anoxic microenvironment, further accelerating the synthesis of lactate [14].

Chronic hepatitis B infection is the primary cause of liver cirrhosis in China [15]. Virus infection can cause hepatocyte inflammation and necrosis, reduced hepatocyte count, and abnormal proliferation of fibrous connective tissue in the liver, destroying normal liver structure and leading to liver dysfunction [16]. Stem cell transplantation is considered as a promising alternative therapy in the research of exploring other effective therapies besides liver transplantation. Many clinical experiments found that MSC treatment improves liver function by increasing ALB secretion, reducing bilirubin level, and enhancing coagulation function [17,18]. In clinical practice, the infusion of mesenchymal stem cells (MSCs) is a safe and short-term efficacious approach to treat patients with end-stage liver disease [17,19]. Among them, the advantages of umbilical cord mesenchymal stem cells (UC-MSCs) in cell acquisition, culture and application make it widely studied. The mechanism of mesenchymal stem cells in the treatment of liver cirrhosis has been controversial. The paracrine effect of MSC is widely studied, which plays the role of anti-oxidation and anti-fibrosis, promoting angiogenesis and cell regeneration by secreting some active cytokines with regulatory effects [20].

Evidence shows that the change in lactate level in the liver is closely related to the degree of liver cirrhosis, and the concentration of extracellular lactate will affect the level of intracellular protein lactylation [1,21]. However, the current research does not focus on the role of liver protein lactate modification in disease development or treatment. We reasonably believe that the changes of protein lactate modification after UC-MSC therapy for liver cirrhosis can provide more microscopic clues for the therapeutic mechanism, which is conducive to the in-depth study of clinical application of UC-MSC. Thus, in this study, we constructed a Kla spectrum focusing on changes in proteomic and protein lactylation modification sites in cirrhosis before and after UC-MSC therapy. After enriching for peptides with differential changes in the liver parenchyma before and after treatment, changes in 160 modification sites of lactylation were identified on 119 proteins. Functional enrichment analysis of the differential modifications revealed that liver glucose metabolism-related enzymes underwent extensive changes in lactylation modification after UC-MSC injection. To the best of our knowledge, this study is the first to comprehensively reveal the changes in protein lactylation modification after UC-MSC therapy at the clinical level. This study may serve as a basis to improve the clinical effects of UC-MSC therapy.

## 2. Materials and Methods

### 2.1. Patients and Study Design

**Figure 1:**
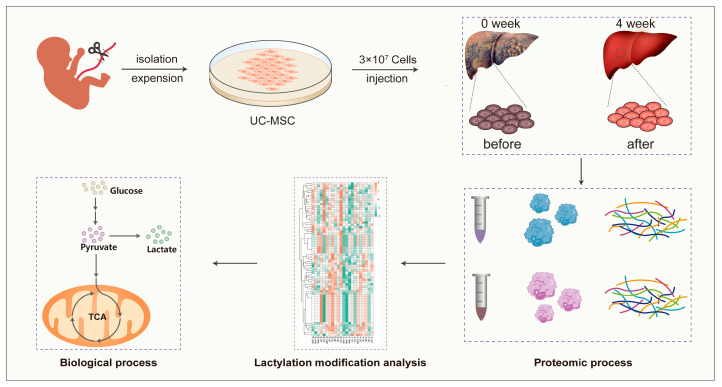
Schematic representation of experimental workflow for analysis characteristics of liver protein lactylation modification before and after treatment with UC-MSCs.

The study protocol conformed to the ethical guidelines of the Declaration of Helsinki and was approved by the Human Ethics Committee of The First Hospital of Lanzhou University, Gansu, China (Ethics number: ldyyky2018−02). The inclusion criteria were as follows: (1) cirrhosis related to hepatitis B viruses and Child-Pugh score B or C, Model for End-Stage Liver Disease ≤ 30; (2) age between 18 and 60; (3) unsatisfactory effect with traditional treatment and refused to undergo liver transplantation; and (4) ALB < 35 g/L, platelet > 30 × 10^9^/L without bleeding tendency, HGB > 70 g/L, HBV DNA< 1.0 × 10^2^ copies/mL. The exclusion criteria were as follows: (1) liver tumor or history of other cancer or pregnancy; (2) treating physician’s decision not to enroll due to severe clinical conditions of the patient; (3) human immunodeficiency virus infection; (4) complications such as gastrointestinal bleeding, hepatic coma, peritonitis, severe feeling, and liver and kidney syndrome within 1 month; (5) portal vein complications, such as portal vein thrombosis, cavernous transformation of portal vein, and Budd-Chiari syndrome; (6) biliary obstruction; and (7) history of splenectomy, portosystemic shunt, and transjugular intrahepatic portosystemic shunt. Each patient was administered UC-MSCs suspended in saline through the portal vein or hepatic artery infusion at a dose of approximately 3 × 10^7^. All patients were followed up for 4 weeks. Data from all patients were collected at baseline and at 1, 2, 3, and 4 weeks after therapy. The levels of some laboratory indicators, including alanine transaminase (ALT), aspartate transaminase (AST), ALB, gamma-glutamyl transferase (GGT), alkaline phosphatase (ALP), total bilirubin (TBIL), indirect bilirubin (IBIL), direct bilirubin (DBIL), cholinesterase (CHE), total cholesterol (TC), TP, hemoglobin (HGB), international normalized ratio (INR), prothrombin activity (PTA), thickness of spleen, splenic vein width, portal vein width, Child-Pugh score, and Meld score were measured to comprehensively evaluate the therapeutic effect of UC-MSCs. Moreover, paired liver biopsies were performed at baseline and 1 month after enrollment (Figure 1).

### 2.2. UC-MSC Isolation, Culture and Identification

UC-MSCs are derived from the umbilical cord of volunteered healthy puerperia from the First Hospital of Lanzhou University. The cell source is provided by Zhongyuan Xiehe cell genetic engineering Co. (Lanzhou, China). and the UC-MSCs are isolated and identified according to the published protocols [22]. The primary UC-MSCs were expanded in vitro, and 3-6 generations of them are used for cell therapy. Moreover, the UC-MSCs were negative for all the tested contaminants before infusion, including Mycoplasma spp., Gram-positive and Gram-negative bacteria, and fungi. The endotoxin levels were below 5 EU/kg, and viability was >80%. All operations comply with the Good Manufacturing Practice of Medical Products (GMP).

### 2.3. Liquid Chromatography−Tandem Mass Spectrometry

The peptides were dissolved in liquid chromatography mobile phase A (0.1% (*v*/*v*) formic acid and 2% (*v*/*v*) acetonitrile) and separated using the EASY−nLC 1200 ultra-performance liquid chromatography (UPLC) system. The liquid phase gradient was set to an increase in the range of 9−22% solvent B (0.1% (*v*/*v*) formic acid in 98% (*v*/*v*) acetonitrile) over 40 min, an increase in the range of 23−32% over 14 min, 80% over 3 min, and a final 80% over 3 min at a constant flow rate of 500 nL/min. The peptides were subjected to nanospray ionization, which was followed by tandem mass spectrometry (MS/MS) in a Q ExactiveTM Plus (Thermo Fisher Scientific, Waltham, MA, USA) coupled online to the UPLC system. The electrospray voltage was 2.1 kV, and the peptide parent ions and their secondary fragments were detected and analyzed by high-resolution Orbitrap. The scanning ranges of primary and secondary MS were set to 350−1400 *m*/*z* and 100 *m*/*z*, respectively, and the scanning resolutions were set to 120,000 and 45,000. To improve the effective utilization of MS and avoid repeated scanning of parent ions, we set the automatic gain control to 1 × 10^5^, the signal threshold to 1 × 10^5^ ions/s, and the maximum implantation time to 100 ms with 15 s dynamic exclusion. 

### 2.4. Database Research

Proteome Discoverer (v2.4.1.15) was used to process the resulting MS/MS data. The tandem mass spectra collected were searched against the Homo_sapiens_9606_SP_20201214.fasta (20,395 sequences) database. Mass errors of fragment ions and the precursor were set as 0.02 Da and 10 ppm, respectively. Trypsin/P was the cleavage enzyme that allowed ≤4 missing cleavages and ≤3 modifications per peptide. Carbamidomethyl (C), TMT16plex (peptide N-terminal), and TMT16plex (K) were set as fixed modifications, and acetyl (protein N-terminal), oxidation (M), and lactylation (K) were set as variable modifications. The minimal peptide was set to six, and the false discovery rate threshold for proteins, modification sites, and peptides was set as 1%. 

### 2.5. Annotation Methods and Functional Enrichment

The Gene Ontology (GO) proteome annotation was derived from the UniProt−GOA database (http://www.ebi.ac.uk/GOA/) (accessed on 3 August 2023). If some identified proteins were not annotated by the UniProt−GOA database, the sequence analysis application InterProScan (v.5.0) was used to annotate the GO functions of the proteins based on protein sequence alignment. Then, the proteins were classified by Gene Ontology annotation based on three categories: biological process, cellular component, and molecular function. The domain functional description of the identified proteins was annotated by InterProScan based on protein sequence alignment, and the InterPro domain database was used. The Kyoto Encyclopedia of Genes and Genomes (KEGG) database was used to annotate protein pathways. The annotation results were mapped on the KEGG pathway database using the KEGG online service tool KEGG mapper. Finally, WoLF PSORT, which is an updated version of PSORT/PSORT II for the prediction of eukaryotic sequences, was used to predict the subcellular localization of differentially expressed proteins. For each category, a two-tailed Fisher’s exact test was employed to test the enrichment of the differentially expressed proteins against all identified proteins. The category with a corrected *p*−value < 0.05 was considered significant. 

### 2.6. Statistical Analysis 

All experiments were set to more than three biological replicates. Continuous variables were expressed as the mean (SD) or median (interquartile range, IQR), and categorical variables were expressed as number. For continuous variables, linear mixed models for repeated measures were used to assess changes from baseline to week 4. For categorical variables, the generalized linear mixed model was used. A two-sided *p*-value of less than 0.05 was considered statistically significant. The analyses were conducted using SAS version 9.4 (SAS Institute, Inc., Cary, NC, USA).

## 3. Results

### 3.1. Patient Characteristics and Therapeutic Effect of UC-MSCs

**Figure 2:**
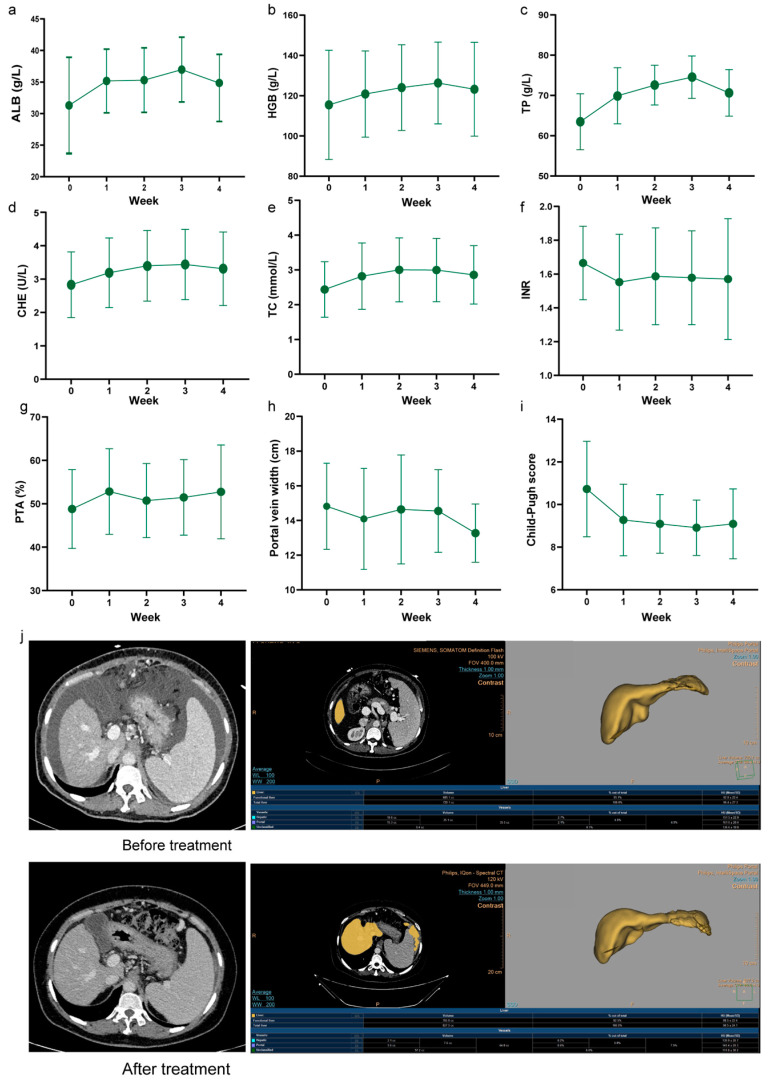
Change characteristics of clinical indicators before and after treatment. (**a**–**i**) ALB, HGB, TP, CHE, TC, INR, PTA, portal vein width, and Child–Pugh score at different time points before and after UC-MSC treatment (*n* = 11). (**j**) Abdominal computed tomography and liver volume modeling diagram of a patient before and after UC-MSC treatment.

A total of 11 patients (nine males and two females) with decompensated hepatitis B virus-related cirrhosis who met the inclusion criteria were enrolled in the study. The baseline clinical parameters of the patients are shown in Table 1. The patients were compared before and after treatment; thus, no control group was needed. The laboratory indexes before and after treatment are shown in Figure 2, and detailed data and *p* values are shown in Table 2. The results showed that the serum ALB of cirrhotic patients showed a steady rising after UC−MSC treatment, and the increase was most obvious in the third week after treatment (week 3 = 37.0, *p* < 0.01) (Figure 2a). HGB also increased significantly after treatment, with an average increase of 10.82 g/L at week 3 after treatment (Figure 2b), and TP also increased most significantly at week 3 (week 4 = 11.05, *p* < 0.001) (Figure 2c). In addition, in the decompensation period of liver function, CHE in patients with cirrhosis decreased. Before treatment, the average of CHE in our enrolled patients was 2.83 ± 0.98, and it increased significantly after treatment, with an average value of 3.31 ± 1.10 (*p* < 0.001) (Figure 2d). Patients with cirrhosis are prone to cholesterol reduction due to a decrease in the ability of the liver to synthesize cholesterol and reserve function, but a sustained significant increase in TC occurred in the second week after UC-MSC treatment (Figure 2e). Although TBIL levels generally decreased, the difference before and after treatment was not significant (week 0 = 62.0, week 4 = 45.8, *p* = 0.62). Coagulation function was also significantly improved, which was manifested by PTA levels significantly increased (week 0 = 48.8 ± 9.1, week 4 = 52.7 ± 10.8, *p* < 0.05); even the average level was still lower than the normal level (Figure 2g). INR decreased significantly (week 0 = 1.67, week 4 = 1.57, *p* = 0.021) (Figure 2f). The width of the portal vein decreased by 1.55 cm on average (*p* < 0.01) (Figure 2h). The Child–Pugh score is an evaluation standard commonly used in the clinic to quantify the liver reserve function of patients with cirrhosis. Four weeks after the end of UC−MSC treatment, the Child–Pugh score of patients was significantly reduced (week 0 = 10.7, week 4 = 9.1, *p* < 0.001), indicating that the liver reserve function was improved to some extent (Figure 2i). In addition, one patient of the enrolled patients had obvious ascites, but these apparently resolved after UC-MSC treatment, and the liver volume increased compared with that before treatment (Figure 2j). These results indicated that the indexes of the patients were better after compared with before treatment. 

### 3.2. Identification and Pattern Analysis of Lysine-Lactylated Sites

Kla is a previously unknown histone modification that has been recently discovered [1]. Changes in histone lactylation modification affect gene expression. However, research on non-histone lactylated proteins is lacking. Therefore, we used the tissue obtained from liver biopsy to degrade trypsin to peptides and analyzed the changes in protein modification. The quality of the identified peptides was checked to confirm the reliability of the MS data. As shown in Figure S1A, most proteins corresponded to more than two peptides, proving the accuracy and credibility of the quantitative results. Afterward, most of the lactylated peptides were distributed in 7–20 amino acids, which accorded with the general law based on enzymatic hydrolysis and MS fragmentation (Figure S1B). The above results suggested that the sample preparation met the standard. In the present study, 7468 proteins were identified, of which 2582 lactylated sites were identified on 722 proteins (Figure S1C,D). These results indicated that the lactic acid modification in the liver cells of patients with liver cirrhosis changed after treatment. On counting the number of protein modification sites, we noticed that 43% (312/723) had one Kla site, 17% (129/723) had two Kla sites, and 8% (63/723) had three Kla sites (Figure S1E). 

**Figure 3:**
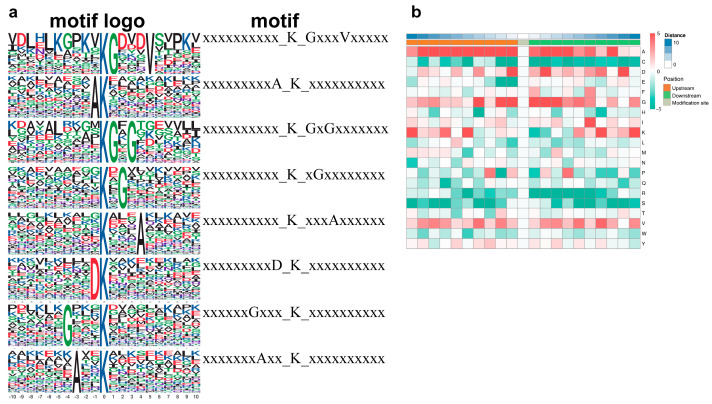
Change characteristics of the Kla peptides in liver after MSC treatment. (**a**) Lactylation sequence motifs for ±10 amino acids surrounding the Kla sites. Lactylation motifs were constructed with Motif−X software (v.1.2). The central K (at position 0) indicates the lactylation lysine. All the surrounding amino acid residues are indicated with the letters in different heights, which is consistent with their frequencies in respective positions. (**b**) Heatmap shows the frequency and degree of change in amino acids near the modification site. Red indicates high frequency and green means low frequency.

The MoMo analysis tool based on motif-x was used to understand the lactylation modification preference for the peptide sequence composed of 10 amino acids, i.e., from −10 to +10 of all identified Kla sites. Based on the analysis results, the amino acid sequences around the lactylation sites were displayed in heatmaps (Figure 3) to determine the flanking sequence of Kla sites. Finally, eight conserved amino acid sequences were extracted and displayed with the WebLogo consensus diagram (Figure 3a). The results showed that certain amino acid residues surrounding the Kla sites were markedly enriched. Residue A was enriched in the −9 to +6 positions; D was enriched in the −1, +2, and +9 positions; G was enriched in the −2 to −1 and +1 to +3 positions; and K was enriched in the −10, −5, +7, and +10 positions (Figure 3b). 

### 3.3. Localization and Functional Enrichment Analysis of Lactylated Proteins 

**Figure 4:**
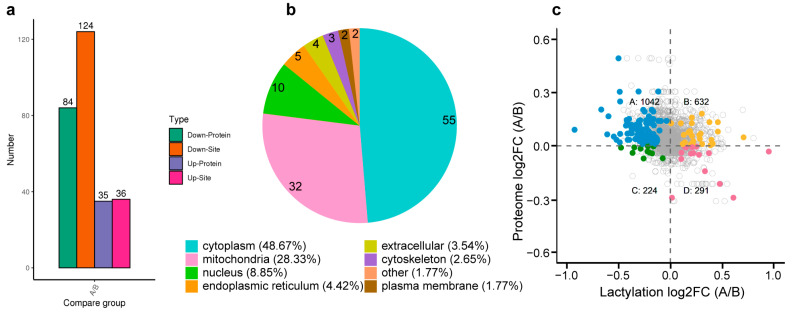
Statistical characteristics of differential lactated proteins. (**a**) Statistics of differential modification sites. (**b**) Subcellular structure annotation classification. (**c**) Four quadrant scatter plots of multiple changes of protein and lactylation sites. The points with color in each quadrant represent changes with statistical differences, and the gray hollow circles represent changes in protein expression and changes in lactylation.

The proteins and the number of lactylation modification sites were compared after and before treatment, and a *p*-value < 0.05 was considered to indicate significance. A fold change (FC) of >1.0 or <1.0 and a *p*-value of <0.05 were used to identify up- and downregulated proteins. Results show that 35 upregulated and 84 downregulated proteins were screened, of which 36 and 124 lactylated sites were upregulated and downregulated after treatment, respectively (Figure 4a). A subcellular localization analysis of the lactylated proteins after treatment was conducted, and results showed that most of them were distributed in the cytoplasm (48%) and mitochondria (28%) (Figure 4b). 

**Figure 5:**
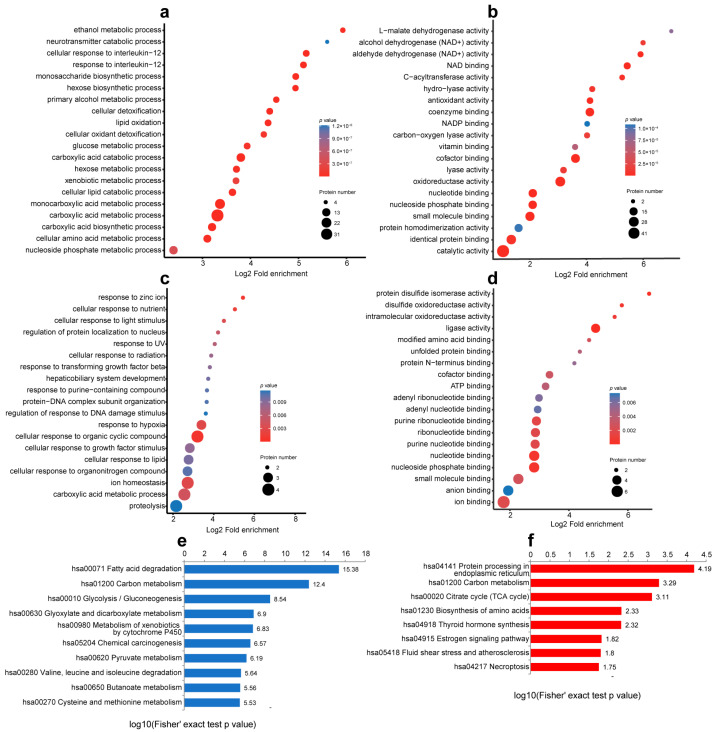
Characteristics of enriched protein. Biological process and molecular function enrichment analysis of downregulated lactated proteins (**a**,**b**) and upregulated lactated proteins (**c**,**d**) based on GO database. (**e**,**f**) represent KEGG pathway of downregulated and upregulated proteins, respectively.

To intuitively observe the changes in protein lactylation modification and expression, we drew a four-quadrant scatter diagram according to the different protein changes and lactylation modifications and counted the number of proteins in each quadrant. Based on the strictly filtered upregulated (D, downregulation of protein expression and upregulation of lactate modification) and downregulated (A, upregulation of protein expression and downregulation of lactate modification) lactylation groups in Figure 4c, we found that 15 Kla sites in 13 proteins were upregulated (FC > 1) and 119 Kla sites in 77 proteins were downregulated (FC < 1) in the liver tissue after compared with before treatment (Figure 4c). GO biological functions and KEGG pathway enrichment maps of these two quadrants are shown in Figure 5. 

The downregulated lactylated proteins in the GO enrichment analysis were involved in metabolic processes, including the metabolism and biosynthesis of various carboxylic acids and the metabolism of cellular amino acids (Figure 5a). Enrichment analysis indicated that the proteins were involved in molecular biological functions, including catalytic and oxidoreductase activities (Figure 5b). The upregulated lactylated proteins were involved in biological processes, such as cell response to organic cyclic compounds and ion balance (Figure 5c). Biomolecular functional analysis suggested that these proteins played roles in the nucleotide binding pathway and ligase activity (Figure 5d). KEGG pathway enrichment analysis showed that the downregulated lactylated proteins were involved in fatty acid degradation (hsa00071), glycolysis and gluconeogenesis (hsa00010), and carbon metabolism (hsa01200) (Figure 5e). The upregulated lactylated proteins were mainly enriched in endoplasmic reticulum protein processing (hsa04141), carbon metabolism (hsa01200), and tricarboxylic acid cycle (hsa00020) pathways (Figure 5f). 

### 3.4. Changes of Glucose Metabolism Related Enzymes Lactylation in Liver

**Figure 6:**
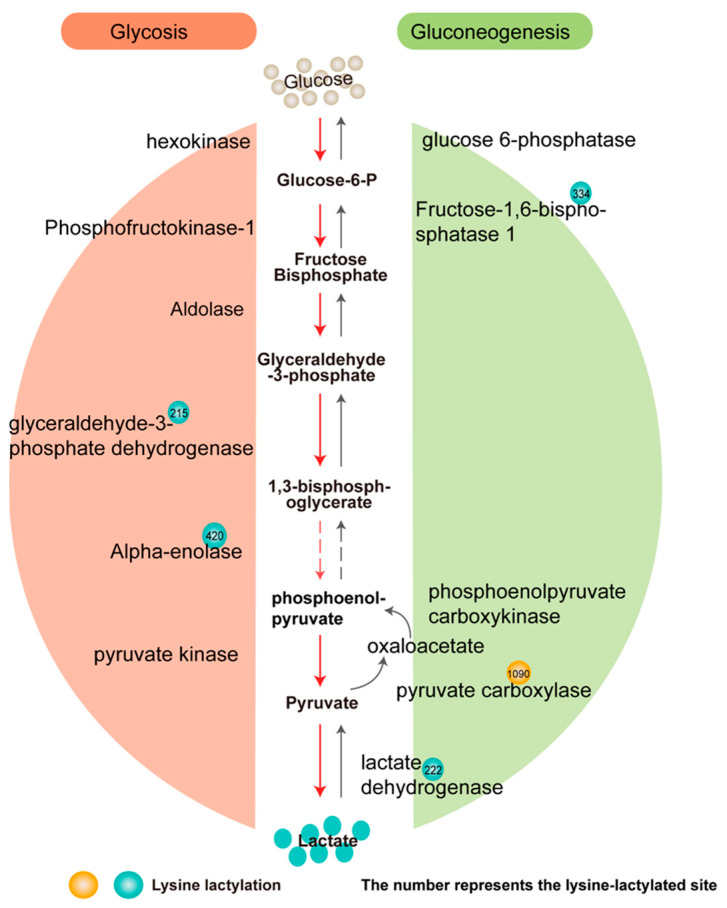
Lactylation modification with statistically significant changes in glycolysis and gluconeogenesis of hepatocytes. The red sphere represents upregulated and the green sphere represents downregulated.

In liver cirrhosis, the glucose metabolism function of hepatocytes is impaired, and the activities of some metabolic enzymes are changed, which may promote the development of liver cirrhosis. Therefore, we focused on the modification of enzymes during glycolysis/gluconeogenesis after UC-MSC treatment. Results showed that a total of 190 Kla sites of 35 proteins involved in metabolism were identified (Table S3), indicating that extensive lactylation changes occurred in metabolic enzymes in the liver after UC-MSC treatment. Through strict screening, one protein is absolutely upregulated and four proteins are absolutely downregulated catalytic enzymes in the sugar metabolism pathway (Figure 6), including glyceraldehyde-3-phosphate dehydrogenase (GAPDH), alpha-enolase (ENO1), fructose-1,6-biophosphatase1 (FBP1), pyruvate carboxylase (PC), and lactate dehydrogenase (LDH). The lysine-lactylated site of LDH occurs at 222 in the L-lactate dehydrogenase A chain, which reversibly catalyzes the conversion of lactic acid and pyruvate [23]. 

### 3.5. Protein Co-Expression Modules Corresponding to Clinical Traits

**Figure 7:**
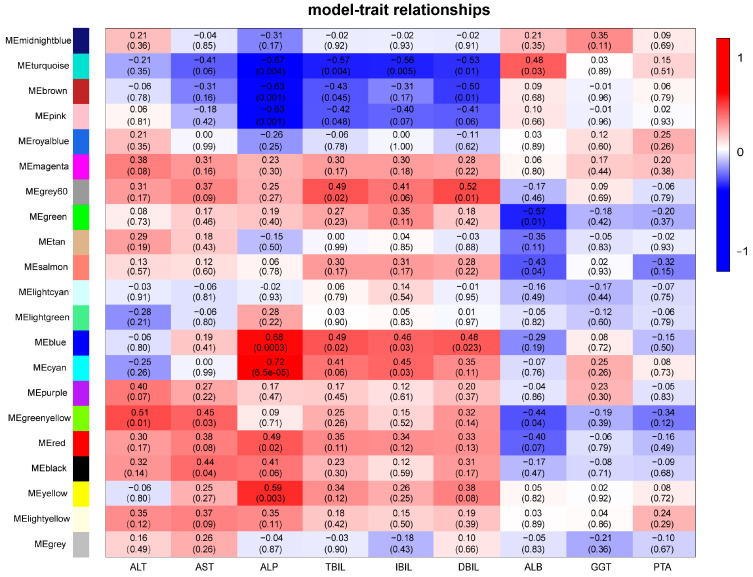
Heatmap of model-trait relationships. Red represents positive correlation and blue represents negative correlation. The values within the lattice represent the correlation coefficient (up) and significance *p* value (down) between the protein and the clinical trait.

Weighted gene co-expression network analysis (WGCNA) is a system biology method used to describe gene association patterns among different samples. Genes with similar expression patterns can be clustered, and the association between modules and specific traits or phenotypes can be analyzed to identify candidate biomarker genes or therapeutic targets [24]. The protein expression profiles of all patients were subjected to WGCNA to identify highly and identically changing protein modules and proteins that may affect changes in clinical indicators after cell therapy according to the association between protein modules and clinical features. A better weight parameter β was selected to establish the adjacency matrix and make the gene distribution conform to the scale-free network according to the connectivity (Figure S2A,B). Cluster analysis was performed on the calculated network topology overlap matrix of power. As shown below, 21 different modules were divided, each of which was indicated by a different color, with gray representing genes that could not be classified in any module (Figure S2C). A heatmap of the relationships between co-expression modules and traits is shown in Figure 7. The turquoise, green–yellow, and blue modules were selected as key modules for further study according to correlation coefficient (r) and *p* values, and those three co-expression modules were associated with multiple clinical traits. The turquoise module was significantly positively correlated with ALB (r = 0.48, *p* = 0.03) but significantly negatively correlated with bilirubin, the index of liver injury (TBIL, r = −0.57, *p* = 0.004; IBIL, r = −0.56, *p* = 0.005; DBIL, r = −0.53, *p* = 0.01). The correlation of the green–yellow module was opposite to that of the turquoise module (ALB, r = −0.44, *p* = 0.04). Meanwhile, the blue module was positively correlated with ALp (r = 0.68, *p* = 0.0003) and bilirubin (TBIL, r = 0.49, *p* = 0.02; IBIL, r = 0.46, *p* = 0.03; DBIL, r = 0.46, *p* = 0.03). These results suggest that some proteins of the above modules are potential factors influencing the clinical treatment effect. Thus, the functional enrichment of these proteins was further analyzed. 

**Figure 8:**
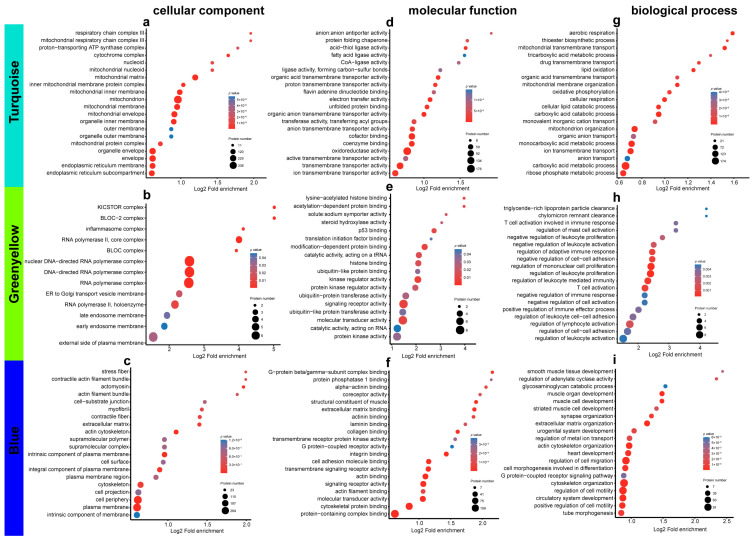
GO analysis involved in turquoise, green–yellow, and blue co-expression module. (**a**–**c**) Cellular component derived from turquoise, green–yellow, and blue co-expression module. (**d**–**f**) Molecular function derived from turquoise, green–yellow, and blue co-expression module. (**g**–**i**) Biological process derived from turquoise, green–yellow, and blue co-expression module.

The GO annotation of the turquoise module showed that in terms of cellular components, its proteins were involved in components of the mitochondria (Figure 8). These proteins were involved in mitochondrial organogenesis and acid metabolic processes, such as the binding of coenzymes and activity of oxidoreductase in terms of molecular biological functions (Figure 8). The GO annotation of the green–yellow module indicated that its proteins were distributed in the RNA polymerase complex, and the molecular biological functions involved were signal receptor activity and kinase regulator activity. These proteins were involved in the regulatory processes of immune cells, including monocyte proliferation, lymphocyte, and T cell activity (Figure 8). The proteins of the blue module and GO enrichment were involved in the composition of cell structures, such as the cell membrane and cytoskeleton. In terms of molecular biological functions, these proteins were involved in the regulation of transmembrane signal receptors and binding of cytoskeleton proteins, including actinin, laminin, and collagen. Therefore, the biological functions included regulating cell migration and cytoskeleton protein generation (Figure 8). The proteins of the turquoise, blue and green–yellow modules were subjected to protein–protein interaction analysis. The proteins were imported into the STRING database with a confidence score > 0.7. The modules with high connectivity in the network were visualized through the MOCDE plug-in for Cytoscape 3.7.2, and then, the possible key biological functions of each module were annotated (Figure S3). 

### 3.6. Proteins Related to Liver Function

**Figure 9:**
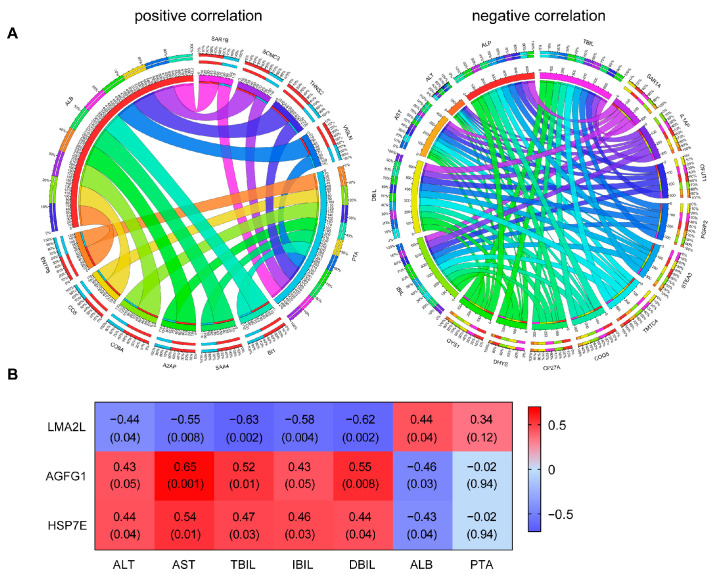
Proteins related to liver function. (**a**) Circos plot. Each of the protein and laboratory indicators is assigned a unique color in the figure, and the arcs depict the correlation between the laboratory indicators and proteins. The larger the circumference of the arc, the more significant the influence of this laboratory indicators on the protein. (**b**) Heatmap of protein–trait relationships. Red represents positive correlation and blue represents negative correlation. The values within the lattice represent the correlation coefficient (up) and significant *p*-value (down) between the protein and the clinical trait.

To understand the clinical significance of protein expression changes and identify the predictive factors after UC-MSC treatment, we divided nine clinical indicators into two categories: positive and negative. The positive indicators, including ALB and PTA, were those indicators whose increase in value after treatment represents an improvement in liver function. The negative indicators were those indicators whose decrease in value after treatment represents an improvement in liver function. Then, the Pearson correlation coefficient was calculated between the two categories of clinical indicators and the relative quantitative values of the proteins in the three modules. In the positive indicators, the top 10 proteins closely related to ALB were identified with r (ALB) > 0, *p* < 0.05, and r (PTA) < 0 as the screening conditions (Table S1). Similarly, among the negative indicators, r (TBIL, IBIL, DBIL) < 0, *p* < 0.05, and r (AST, ALT) < 0 were used as the screening conditions to identify the top 10 proteins closely related to bilirubin (the absolute value of the correlation coefficient was the largest) (Table S2). The final protein was obtained using the Circos website (http://circos.ca/) (accessed on 3 August 2023) online platform (http://mkweb.bcgsc.ca/tableviewer/) (accessed on 3 August 2023) to draw the positive and negative correlation circles (Figure 9a). Interestingly, when we combined the two screening conditions, three proteins, LMAN2L, AGFG1, and HSPA14, were obtained (Figure 9b). LMA2L was negatively correlated with AST, ALT, ALP, TBIL, IBIL, DBIL, and other indicators of liver injury but positively correlated with ALB, PTA, and other indicators of liver function recovery; thus, it can be used as a positive predictor. AGFG1 and HSPA14 were positively correlated with ALT, AST, TBIL, IBIL, DBIL, and other indicators of liver injury but negatively correlated with ALB, PTA, and other indicators of liver function recovery; thus, they can be used as negative predictors (Figure 9b). 

## 4. Discussion

The use of UC-MSCs to treat liver fibrosis/cirrhosis has attracted increasing attention because of the convenient storage and low immunogenicity of these cells [25,26]. The results of the present study were consistent with those of previous studies. The patients in the present study had increased the synthesis and secretion of ALB, TP, and HGB, improved coagulation function, and significantly increased PTA and considerably decreased TBIL. An experiment using an animal model of liver cirrhosis induced by CCl4 showed that UC-MSC improved liver function and reduced mortality by regulating the immune microenvironment dominated by chronic liver inflammation and hepatic stellate cells, which are the main effector cells of liver fibrosis [27]. Some cytokines (human growth factor, vascular endothelial growth factor) secreted by UC-MSCs can activate cell proliferation signals and promote liver regeneration [28]. However, the results obtained from animal models are difficult to verify at the clinical level because of ethical concerns. The main objective of the present study was to elucidate the therapeutic mechanism of UC-MSCs by combining proteomic and modified-omics analyses and identify predictors of treatment through the co-expression analysis of some proteins significantly correlated with clinical traits. 

The liver plays a role in metabolism, synthesis, and detoxification. Therefore, the normal glucose metabolism of the liver is important to maintain a relatively constant blood glucose level and normal energy supply of the body [29]. The metabolic function of hepatocytes changes in liver cirrhosis, which is characterized by insulin resistance and increased gluconeogenesis [30]. Liver cirrhosis can enhance the aerobic glycolysis of hematopoietic stem cells combined with the insufficient tissue perfusion resulting from vascular system disorder, leading to low lactate clearance and increased lactate levels in the liver and serum of patients with liver cirrhosis [1,11,14]. Lactate is a lactylation donor. Lactate is closely related to histone lactylation, which is a new post-translational modification found by Zhao et al. [1]. It can promote the transformation of M1 to the M2 phenotype by initiating the expression of homeostatic genes associated with M2-like macrophages [1]. In ocular melanoma, histone lactylation regulates tumorigenesis through crosstalk with RNA modification [31]. However, beyond histones, a few studies focused on a wide range of proteins with broad Kla sites. Therefore, in the present study, we systematically studied the changes in lactylation modification after UC-MSC treatment of liver cirrhosis. Results showed that a wide range of lactylation modification changes occurred in intracellular proteins after treatment, especially in enzymes associated with metabolism. We enriched differentially expressed proteins in glucose metabolism (map00010 and map00620), amino acid metabolism (map00270), and fatty acid metabolism (map00071). Most of the enzymes of glucose metabolism/gluconeogenesis and citric acid cycle in the liver have undergone changes in lactate modification after UC-MSC treatment, which may be a positive signal, suggesting that we should pay attention to the roles of lactate modification changes in the process of liver recovery.

The level of protein lactylation modification generally decreased. In particular, 36 lactylated sites were upregulated, and 124 lactylated sites were downregulated. This result may be related to the lactate level reduction in the liver microenvironment after UC-MSC therapy. These changes may be ascribed to different reasons. First, UC-MSC therapy may improve the glucose metabolism of hepatocytes, promote oxidative phosphorylation, and reduce the transformation from pyruvate to lactate. Second, UC-MSC therapy may improve the structural disorder of liver cirrhosis, alleviate tissue hypoxia, and then reduce the lactate production of anaerobic glycolytic by enhancing cell oxygen supply. Third, UC-MSCs may promote the regeneration of hepatocytes following the improvement of a severe microenvironment of cirrhosis and enhance the reuse of lactate. Unfortunately, we did not verify the reasons for the changes because of the lack of additional organizations, which is one of the limitations of this work. 

WGCNA can enrich proteins that are related to each other in biological functions, which are called protein modules. The correlation between the proteome and clinical traits can be studied through the co-expression modules. With this process, key proteins that can be used as biomarkers for diagnosis or treatment can be easily identified [6]. In the present study, we selected liver tissue samples from patients with liver cirrhosis before and after treatment. A total of 22 proteomic samples and 9 clinical traits were used to draw the module–trait relationship heatmap, and three key modules were further analyzed. Ten proteins, namely, THNS2, CO8A, SCMC3, ENTP5, SAR1B, A2AP, SAA4, CO5, VIGLN, and BI-1, were positively correlated with ALB and PTA, which are usually used to evaluate the improvement of liver function. Among of them, BI-1 (Bax inhibitor-1) is an inhibitor of Bax-induced apoptosis that is involved in cytoprotective functions, including reactive oxygen species regulation and endoplasmic reticulum stress [32]. The expression of the bi-1 gene in the liver of mice decreases after CCl4-induced liver fibrosis but increases again after MSC treatment [33]. In our study, after MSC therapy, the expression of the BI-1 protein in patients with elevated ALB also increased, and the two were positively correlated (r = 0.46 *p* = 0.03). The change in BI-1 protein can be the result of cell therapy, and it can also be the mechanism, which seems to suggest that the MSC therapy improves liver function by reducing hepatocyte apoptosis. In addition, 10 clinical characteristics were screened alongside bilirubin and transaminase. The levels of these indicators would increase in the case of liver injury and negatively correlate with TMTC4, STEA3, DHYS, COQ5, PGRP2, IL1AP, OFUT1, SAR1A, CP27A, and GYS1. Previous studies rarely discussed the correlation between these proteins and the liver therapeutic effect. The clinical values of these proteins warrant further investigation. The changes of these proteins have a close correlation with the evaluation indicators of liver function, which may give us a new hint that these proteins may affect the recovery of liver function and become potential therapeutic targets. However, there is not enough evidence at present.

## 5. Conclusions

This study comprehensively revealed the changes in protein modification and expression after UC-MSC treatment of liver cirrhosis through the liver biopsy of patients combined with protein lactylation and proteomics. Results showed that lactylation modification changes widely occurred after cell treatment, especially in enzymes related to glucose metabolism. This result indicated that cell therapy affected the metabolic function of hepatocytes and regulated cell function. Through WGCNA, we identified proteins that were closely related to clinical liver function indicators, and these proteins could serve as potential predictors of the efficacy of stem cells in the treatment of liver cirrhosis. However, an obvious limitation of this work was the lack of result verification because of the rarity of samples. In specific, the changes in lactate level in the liver microenvironment after treatment and the effect of the lactylation modification of enzymes on metabolic function warrant verification. Based on the current findings, our future studies will focus on revealing the potential mechanisms of UC-MSC in the treatment of liver cirrhosis from the perspective of glucose metabolism.

## Figures and Tables

**Table 1:** Basic characteristics of patients.

Variables	UC-MSCs Treatment (*n* = 11)
Sex (male/female)	9/2
Age (years)	41.2 ± 9.3
BMI (kg/m^2^)	24.3 ± 3.5
Diabetes	0
Hypertension	0
Cyst	6
Gallstone	1
Pleural effusion	2

Data are presented mean ± SD or number.

**Table 2:** Clinic characteristics of patients.

Variables	Week 0	Week 1	Week 2	Week 3	Week 4
ALT	35.6 ± 21.4	30.3 ± 10.9	33.7 ± 12.6	35.6 ± 17.8	33.1 ± 10.7
AST	68.3 ± 63.8	57.7 ± 20.0	64.5 ± 30.4	60.4 ± 23.6	54.5 ± 15.3
ALB	31.3 ± 7.6	35.2 ± 5.0	35.3 ± 5.1 *	37.0 ± 5.1 *	34.8 ± 5.5 **
GGT	51.9 ± 44.6	59.6 ± 38.5	53.0 ± 28.4	49.0 ± 23.5	43.5 ± 22.6
ALP	108.0 ± 53.1	122.1 ± 67.5	124.9 ± 64.2	122.9 ± 69.0	115.0 ± 56.0
TBIL	62.0 (35.4−81.9)	51.9 (39.2−78.7)	49.3 (38.7−89.2)	54.3 (39.0−91.4)	45.8 (31.1−66.5)
IBIL	39.2 ± 17.6	40.1 ± 16.0	46.2 ± 25.3	45.6 ± 20.8	39.2 ± 23.4
DBIL	24.5 ± 20.6	23.8 ± 21.4	25.0 ± 26.7	23.9 ± 25.9	21.5 ± 27.4
CHE	2.83 ± 0.98	3.19 ± 1.04	3.40 ± 1.06 **	3.44 ± 1.05 ***	3.31 ± 1.10 ***
TC	2.44 ± 0.80	2.82 ± 0.95	3.00 ± 0.92 **	3.00 ± 0.91 ***	2.86 ± 0.84 ***
TP	63.5 ± 7.0 **	69.9 ± 7.0 ***	72.5 ± 4.9 ***	74.5 ± 5.3 ***	70.6 ± 5.8 ***
HGB	115.5 ± 27.1	120.8 ± 21.4	124.0 ± 21.3	126.3 ± 20.3 **	123.2 ± 23.3 **
INR	1.67 ± 0.22	1.55 ± 0.28	1.59 ± 0.29	1.58 ± 0.28	1.57 ± 0.36
PTA	48.8 ± 9.1	52.8 ± 9.9	50.7 ± 8.5	51.5 ± 8.7	52.7 ± 10.8 *
Thickness of Spleen	58.9 ± 13.1	62.5 ± 12.3	59.2 ± 13.1	62.0 ± 11.7	63.4 ± 13.5 *
Splenic vein width	10.18 ± 2.89	10.00 ± 2.86	11.00 ± 3.29	11.34 ± 3.27	11.00 ± 3.10
Portal vein width	14.82 ± 2.48	14.09 ± 2.91	14.64 ± 3.14	14.55 ± 2.38	13.27 ± 1.68 **
Child−Pugh score	10.7 ± 2.2	9.3 ± 1.7	9.1 ± 1.4 **	8.9 ± 1.3 **	9.1 ± 1.6 ***
Meld score	13.94 ± 4.03	14.10 ± 4.03	15.57 ± 4.32	15.27 ± 4.33	13.95 ± 4.26

Data are presented mean ± SD or median (IQR). * *p* < 0.05 compared with week 0. ** *p* < 0.01 compared with week 0. *** *p* < 0.001 compared with week 0.

## Data Availability

All proteomics data in the manuscript are available via ProteomeXchange with identifier PXD037530.

## Supplementary Materials

The following supporting information can be downloaded at https://www.mdpi.com/article/10.3390/cimb45100532/s1, Supplementary methods. Figure S1: Overview of lysine lactylation sites and proteins identification; Figure S2: (A) Determination of soft threshold (power). (Left) Scale-free topology fit index as a function of different soft threshold (power), the line represents that R2 = 0.9; (Right) Mean connectivity as a function of different soft threshold (power). (B) Check scale-free topology, and here the adjacency matrix was defined using soft-thresholds with beta = 0.9 for mRNA data. (C) Module hierarchical clustering tree and module profiles. The color row underneath the dendrogram shows the module assignment determined by the Dynamic Tree Cut; Figure S3: Protein–protein interaction network of module protein; Table S1: Summary of top 10 proteins closely related to ALB; Table S2: Summary of top 10 proteins closely related to bilirubin; Table S3: Summary of lactated protein associated with glycolysis/gluconeogenesis.
