# Supplementary material for: Protein Lactylation Modification and Proteomics Features in Cirrhosis Patients after UC-MSC Treatment
Source: Curr Issues Mol Biol. 2023 Oct 18;45(10):8444–60. doi: 10.3390/cimb45100532 (PMC10605309; doi:10.3390/cimb45100532)
Supplement: Supplementary file 1 [file cimb-45-00532-s001.zip › cimb-2650871-supplementary.pdf]

Supplementary materials and methods

1. Protein extraction and trypsin digestion

Liver biopsy samples were taken out from -80℃ and added liquid nitrogen and fully grinded into powder. Each sample were added with 4 volumes of powder lysis buffer (1% TritonX-100, 1% protease inhibitor, 3μM TSA, 50 mM Nicotinamide (NAM, Sigma, USA)), ultrasonic lysis. Then, the mixture was centrifuged at 12000g at 4◦C for 10 min for removing cell fragments. Finally, the supernatant was transferred into a new centrifuge tube, and the protein concentration was determined with a bicinchoninic acid kit (Beyotime Institute of Biotechnology, China). Take an equal amount of each sample protein for enzymatic hydrolysis, add an appropriate amount of standard protein, and adjust the volume to the same with lysate. TCA was added slowly into the sample to 20%. After vortex mix, the mixture was precipitated at 4℃ for 2h and then centrifuged at 4500g for 5min. The supernatant was discarded and the precipitated was washed with precooled acetone for 2-3 times. TEAB (Sigma–Aldrich, USA) was added into the precipitation at a final concentration of 200 mM following dispersing by ultrasound. Trypsin (Promega, Madison, WI, USA) was added at a 1:50 trypsin-to-protein mass ratio, and the digestion was performed overnight. Dithiothreitol (DTT, Sigma, USA) was added to make the final concentration of 5 mM and reduced at 37 ℃ for 60 min. Then, iodoacetamide (IAA, Sigma–Aldrich, USA) was added at the final concentration of 11 mM and incubated at room temperature in the dark for 45 min.

2. TMT lable, HPLC fractionation and modify enrichment

The peptides were desalted with Strata X C18 (Phenomenex) and vacuum freeze-dried. The peptides were dissolved with 0.5M TEAB and labeled TMT according to the operation instructions. Briefly, the labeled reagent was dissolved in acetonitrile after thawing. The mixture added with the peptide

and incubated at room temperature for 2 hours. After that, the mixed labeled peptides were desalted and vacuum freeze-dried. The labeled peptides (6 mg) were separated into fractions by high-pH, reverse-phase High-performance liquid chromatography (HPLC) on an Agilent 300Extend C18 (5 μm particles, 4.6 mm inside diameter, 250 mm length; Thermo Fisher Scientific, Waltham, MA, United States). Briefly, peptides were separated into 60 fractions using an acetonitrile gradient of 8–32% acetonitrile (pH 9.0) over 60 min. The peptides were then combined into two fractions and dried by vacuum centrifugation. Peptides dissolved in IP buffer (100 mM NaCl, 1 mm EDTA, 50 mm Tris HCl, 0.5% NP-40, pH 8.0) were incubated with pre-washed lactic acid resin (PTM-1404, Hangzhou Jingjie Biotechnology Co., Ltd., China) overnight at 4℃ with gentle shaking. After incubation, the resin was washed with IP buffer solution for four times and with deionized water for twice. Finally, 0.1% trifluoroacetic acid eluent was used to elute the resin bound peptides for three times. For the LC-MS/MS analysis, the peptides were desalted with C18 ZipTips (Merck Millipore, USA) according to the manufacturer's instructions.

3. Enrichment-based clustering

For further hierarchical clustering based on differentially expressed protein functional classification (such as: GO, Domain, Pathway, Complex). We first collated all the categories obtained after enrichment along with their P values, and then filtered for those categories which were at least enriched in one of the clusters with P value <0.05. This filtered P value matrix was transformed by the function x = −log10 (P value). Finally, these x values were z-transformed for each functional category. These z scores were then clustered by one-way hierarchical clustering (Euclidean distance, average linkage clustering) in Genesis. Cluster membership were visualized by a heat map using the "heatmap.2" function from the "gplots" R-package.

4. Protein-protein interaction network

All differentially expressed protein database accession or sequence were searched against the STRING database version 11.0 for protein-protein interactions. Only interactions between the proteins belonging to the searched data set were selected, thereby excluding external candidates. STRING defines a metric called “confidence score” to define interaction confidence; we fetched all interactions that had a confidence score ≥ 0.7 (high confidence). Interaction network form STRING was visualized in R package “networkD3”.

Supplementary figures and tables

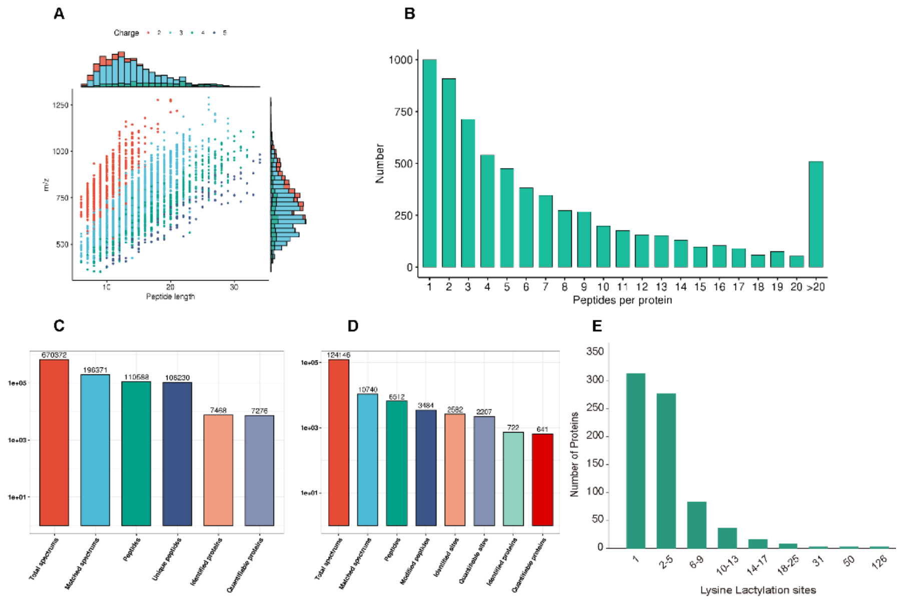

Figure S1. overview of lysine lactylation sites and proteins identification. (A) Schematic diagram of peptide length distribution. (B) Peptide number distribution. (C) and (D)The total number of identified peptides and proteins after mass spectrometry data filtering. (E) Statistics of all lactation modification sites on a protein.

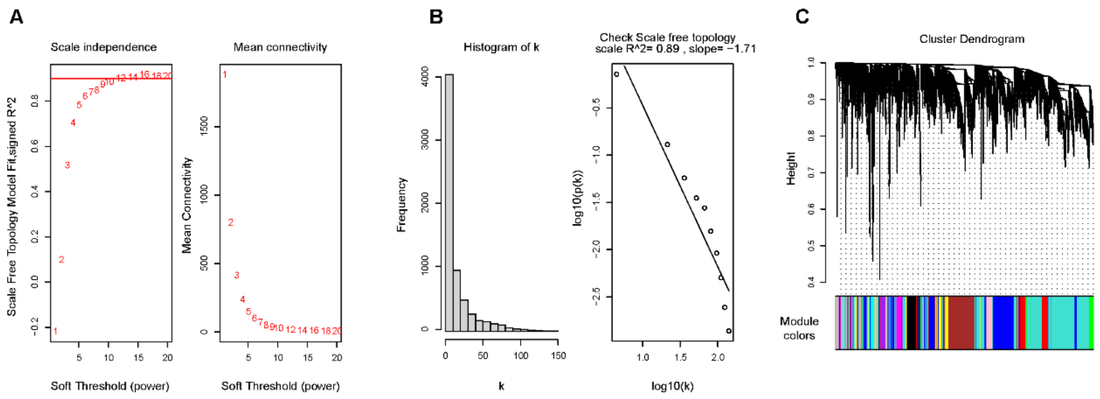

Figure S2. (A) Determination of soft threshold (power). (Left) Scale-free topology fit index as a function of different soft threshold (power), the line represents that R2 = 0.9; (Right) Mean connectivity as a function of different soft threshold (power). (B) Check scale-free topology, and here the adjacency matrix was defined using soft-thresholds with beta = 0.9 for mRNA data. (C) Module hierarchical clustering tree and module profiles. The color row underneath the dendrogram shows the module assignment determined by the Dynamic Tree Cut.

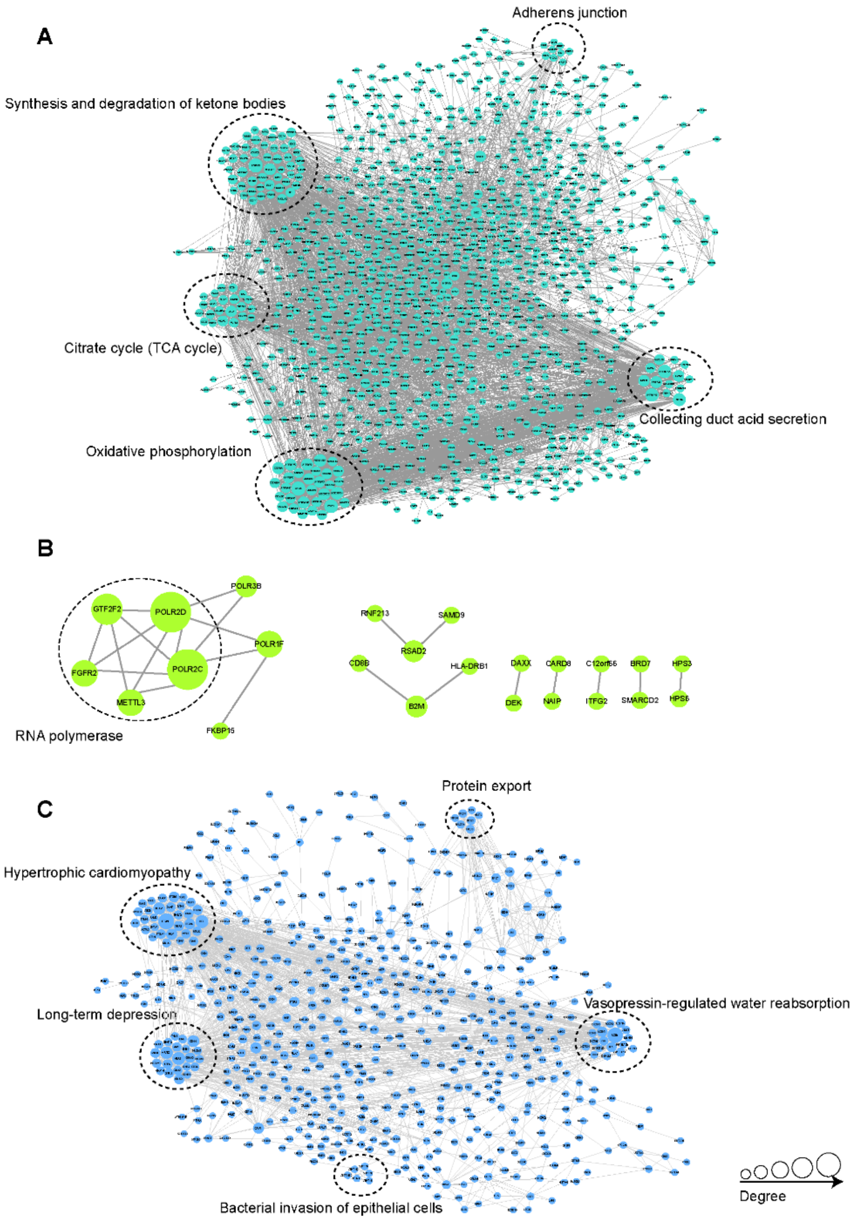

Figure S3 Protein–protein interaction network of module protein. (A), (B), and (C) is the network interaction diagram of the three module proteins of turquoise, greenyellow and blue. The larger the circle, the more interaction nodes. Show the gene name in the circle. Marks the top 1 KEGG pathway in which each module.

Supplementary Table S1 Summary of top 10 proteins closely related to ALB

Protein accession	Gene name	ALB	PTA	ALT	AST	ALP	TBIL	IBIL	DBIL
Q86YJ6	THNS2	0.43	0.56	0.48	0.06	-0.38	-0.40	-0.30	-0.45
		0.05	0.01	0.02	0.78	0.08	0.07	0.18	0.04
P07357	CO8A	0.43	0.55	-0.17	-0.28	-0.17	-0.38	-0.33	-0.39
		0.05	0.01	0.45	0.21	0.44	0.08	0.13	0.07
Q9BV35	SCMC3	0.44	0.50	0.03	-0.21	-0.42	-0.48	-0.46	-0.45
		0.04	0.02	0.90	0.35	0.05	0.02	0.03	0.03
O75356	ENTP5	0.45	0.44	-0.16	-0.41	-0.52	-0.55	-0.46	-0.57
		0.04	0.04	0.49	0.06	0.01	0.01	0.03	0.01
Q9Y6B6	SAR1B	0.45	0.44	-0.11	-0.35	-0.53	-0.51	-0.42	-0.55
		0.04	0.04	0.63	0.11	0.01	0.02	0.05	0.01
P08697	A2AP	0.45	0.43	-0.04	-0.27	-0.28	-0.36	-0.25	-0.42
		0.03	0.04	0.85	0.22	0.21	0.10	0.27	0.05
P35542	SAA4	0.46	0.58	-0.05	-0.41	-0.46	-0.51	-0.32	-0.62
		0.03	0.004	0.84	0.06	0.03	0.02	0.15	0.002
P01031	CO5	0.46	0.57	-0.19	-0.39	-0.25	-0.65	-0.55	-0.67
		0.03	0.01	0.41	0.07	0.26	0.001	0.01	0.0006
Q00341	VIGLN	0.47	0.44	0.17	-0.18	-0.51	-0.42	-0.37	-0.43
		0.03	0.04	0.44	0.42	0.01	0.05	0.09	0.05

P55061	BI1	0.47	0.46	0.28	-0.03	-0.41	-0.33	-0.34	-0.30
		0.03	0.03	0.21	0.91	0.06	0.13	0.12	0.17

The upper row represents the correlation coefficient, and the lower row represents the *p*-value.

Supplementary Table S2 Summary of top 10 proteins closely related to bilirubin

Protein accession	Gene name	ALB	PTA	ALT	AST	ALP	TBIL	IBIL	DBIL
Q5T4D3	TMTC4	0.30	0.41	-0.30	-0.58	-0.56	-0.76	-0.68	-0.76
		0.17	0.06	0.18	0.005	0.01	0.00004	0.001	0.00004
Q658P3	STEA3	0.52	0.32	-0.15	-0.40	-0.70	-0.72	-0.67	-0.70
		0.01	0.14	0.51	0.07	0.0003	0.0002	0.0007	0.0003
P49366	DHYS	0.33	0.27	-0.10	-0.20	-0.52	-0.69	-0.66	-0.67
		0.13	0.22	0.66	0.37	0.01	0.0003	0.0009	0.0007
Q5HYK3	COQ5	0.36	0.16	-0.05	-0.33	-0.79	-0.69	-0.68	-0.64
		0.10	0.47	0.82	0.14	0.00001	0.0004	0.0005	0.001
Q96PD5	PGRP2	0.54	0.39	-0.18	-0.58	-0.66	-0.69	-0.55	-0.74
		0.01	0.08	0.41	0.005	0.001	0.000	0.01	0.0001
Q9NPH3	IL1AP	0.22	0.02	-0.27	-0.43	-0.62	-0.68	-0.71	-0.58
		0.32	0.92	0.22	0.05	0.002	0.001	0.0002	0.004
Q9H488	OFUT1	0.62	0.32	-0.19	-0.47	-0.62	-0.67	-0.61	-0.67
		0.002	0.15	0.40	0.03	0.002	0.001	0.003	0.001
Q9NR31	SAR1A	0.54	0.47	-0.01	-0.39	-0.70	-0.67	-0.56	-0.71
		0.01	0.03	0.98	0.07	0.0003	0.0006	0.01	0.0002
Q02318	CP27A	0.31	0.16	-0.20	-0.39	-0.70	-0.67	-0.69	-0.60
		0.16	0.46	0.37	0.07	0.0003	0.0006	0.0004	0.003
P13807	GYS1	0.23	0.02	-0.24	-0.43	-0.74	-0.67	-0.63	-0.64

	0.31	0.93	0.27	0.05	0.0001	0.0007	0.002	0.001

The upper row represents the correlation coefficient, and the lower row represents the *p*-value.

Supplementary Table S3 Summary of lactated protein associated with glycolysis/gluconeogenesis

protein accession	protein description	gene names	Kla position	count
P07327	Alcohol dehydrogenase 1A	ADH1A	234, 248, 340, 249, 227, 367, 339, 114, 20, 105, 331, 324, 326, 100	14
P00325	All-trans-retinol dehydrogenase [NAD (+)] ADH1B	ADH1B	234, 331, 248, 324, 136, 340, 20, 33, 249, 227, 326, 339, 114, 326, 316, 105, 100	17
P00326	Alcohol dehydrogenase 1C	ADH1C	234, 248, 249, 227, 339, 114, 100, 331, 20, 105	10
P08319	All-trans-retinol dehydrogenase [NAD (+)] ADH4	ADH4	20, 33, 239, 254, 345, 234, 120, 342, 132, 336	10
P11766	Alcohol dehydrogenase class-3	ADH5	284	1
P28332	Alcohol dehydrogenase 6	ADH6	17, 232, 227	3
P14550	Aldo-keto reductase family 1 member A1	AKR1A1	127, 85, 287, 308	4
P30837	Aldehyde dehydrogenase X, mitochondrial	ALDH1B1	426, 383, 500, 399, 52, 414, 379, 429, 364	9
P05091	Aldehyde dehydrogenase, mitochondrial	ALDH2	428, 369, 52, 378, 383, 368, 73, 511, 355, 159, 155	11
P51648	Aldehyde dehydrogenase family 3 member A2	ALDH3A2	324	1

P49419	Alpha-aminoadipic semialdehyde dehydrogenase	ALDH7A1	537, 439, 93, 389, 390	5
P49189	4-trimethylaminobutyraldehyde dehydrogenase	ALDH9A1	303, 30, 49, 298,310	5
P04075	Fructose-bisphosphate aldolase A	ALDOA	147, 342, 312, 230, 108, 42, 14	7
P05062	Fructose-bisphosphate aldolase B	ALDOB	321, 242, 317, 340, 147, 101, 13, 108, 140, 243, 49, 99	13
P09972	Fructose-bisphosphate aldolase C	ALDOC	108, 147, 42	3
P10515	Dihydrolipoyllysine-residue acetyltransferase component of pyruvate dehydrogenase complex, mitochondrial	DLAT	473	1
P09622	Dihydrolipoyl dehydrogenase, mitochondrial	DLD	430, 267, 159, 166, 66	5
P06733	Alpha-enolase	ENO1	71, 64, 81, 330, 92, 420, 233, 80, 202, 60, 28, 193	12
P13929	Beta-enolase	ENO3	28	1
P09467	Fructose-1,6-bisphosphatase 1	FBP1	110, 101, 151, 334	4
O00757	Fructose-1,6-bisphosphatase isozyme 2	FBP2	208	1
Q96C23	Galactose mutarotase	GALM	101	1
P04406	Glyceraldehyde-3-phosphate dehydrogenase	GAPDH	117, 219, 215, 251, 271, 194, 263, 139	8
P06744	Glucose-6-phosphate isomerase	GPI	234, 252, 447, 454	4

Q2TB90	Hexokinase HKDC1	HKDC1	527	1
P00338	L-lactate dehydrogenase A chain	LDHA	14, 318, 243, 222, 81	5
Q16822	Phosphoenolpyruvate carboxykinase [GTP], mitochondrial	PCK2	294, 578, 489, 108	4
P17858	ATP-dependent 6-phosphofructokinase, liver type	PFKL	392, 397, 677	3
P08237	ATP-dependent 6-phosphofructokinase, muscle type	PFKM	678	1
P18669	Phosphoglycerate mutase 1	PGAM1	106, 251, 113, 100, 253	5
P15259	Phosphoglycerate mutase 2	PGAM2	251, 100	2
P00558	Phosphoglycerate kinase 1	PGK1	216,131, 192, 323, 91, 220, 141, 291	8
P36871	Phosphoglucomutase-1	PGM1	457, 164, 16, 349	4
P14618	Pyruvate kinase PKM	PKM	247	1
P60174	Triosephosphate isomerase	TPI1	142, 194, 19, 69, 188, 175	6
